# The role of blood groups, vaccine type and gender in predicting the severity of side effects among university students receiving COVID-19 vaccines

**DOI:** 10.1186/s12879-023-08363-0

**Published:** 2023-06-06

**Authors:** Ohoud S. Almalki, Eman Y. Santali, Abdulaziz A. Alhothali, Ashraf A. Ewis, Abeer Shady, Ahmed Ibrahim Fathelrahman, Sayed F. Abdelwahab

**Affiliations:** 1 Department of Clinical Pharmacy, College of Pharmacy, Taif University, Taif, 21944 Saudi Arabia; 2 Department of Pharmaceutical Chemistry, College of Pharmacy, Taif University, Taif, 21944 Saudi Arabia; 3 College of Pharmacy, Taif University, Taif, 21944 Kingdom of Saudi Arabia; 4 Department of Public Health and Occupational Medicine, Faculty of Medicine, Minia University, Minia, 61511 Egypt; 5 Department of Public Health, Faculty of Health Sciences-AlQunfudah, Umm Al-Qura University, Makkah, 28821 Saudi Arabia; 6 Department of Pharmaceutics and Industrial Pharmacy, College of Pharmacy, Taif University, Taif, 21944 Saudi Arabia

**Keywords:** COVID-19, Predictors, Saudi Arabia, Side effects, Severity, Vaccines, Young

## Abstract

On March 11^th^, 2020, the World Health Organization (WHO) declared the coronavirus disease 2019 (COVID-19) a pandemic. To control the pandemic, billions of vaccine doses have been administered worldwide. Predictors of COVID-19 vaccine-related side effects are inconsistently described in the literature. This study aimed to identify the predictors of side effects’ severity after COVID-19 vaccination among young adult students at Taif University (TU) in Saudi Arabia. An online, anonymous questionnaire was used. Descriptive statistics were calculated for numerical and categorical variables. Possible correlations with other characteristics were identified using the chi-square test. The study included 760 young adult participants from TU. Pain at the injection site (54.7%), headache (45.0%), lethargy and fatigue (43.3%), and fever (37.5%) were the most frequently reported COVID-19 vaccine-related side effects after the first dose. The most frequent side effects were reported among the 20–25-year-old age group for all doses of all vaccines. Females experienced remarkably more side effects after the second (*p* < 0.001) and third doses (*p* = 0.002). Moreover, ABO blood groups significantly correlated with vaccine-related side effects after the second dose (*p* = 0.020). The participants' general health status correlated with the side effects after the first and second doses (*p* < 0.001 and 0.022, respectively). The predictors of COVID-19 vaccine-related side effects in young, vaccinated people were blood group B, female gender, vaccine type, and poor health status.

**Supplementary Information:**

The online version contains supplementary material available at 10.1186/s12879-023-08363-0.

## Introduction

Recently, the world has been facing one of the most widespread and significant public health crises due to the novel coronavirus disease 2019 (COVID-19) that is caused by severe acute respiratory syndrome coronavirus 2 (SARS-CoV-2). On March 11^th^, 2020, the World Health Organization (WHO) [1] declared COVID-19 a pandemic. Worldwide spread was rapid, and each infection presents an opportunity for new virus mutations. The pandemic’s social, economic, and psychological impacts are devastating [2, 3]. Two years into the pandemic, the coronavirus has now confirmed more than 5.5 million deaths worldwide, according to data from Johns Hopkins University (February 15^th^, 2022). Life had come to a standstill, and there was an urgent need to find a way back to normal life and prevent rapid spread of the virus, which could lead to hospitalization, intubation, and possibly death from acute respiratory distress syndrome. Vaccination is an important safeguard against COVID-19 infection [4] and plays an important role in boosting population immunity, preventing severe illness, and mitigating health crises [5]. To face the pandemic, Saudi Arabia’s government adopted several public health policies, including vaccination, lockdown, enforcement of social distancing, virtual learning in educational institutions, and home delivery of health services.

After the WHO declared COVID-19 a pandemic in March 2020, the Food and Drug Administration entities in different countries issued an emergency use authorization (EUA) for COVID-19 vaccines [6] for individuals who are 18 years and older [6, 7]. The first COVID-19 vaccine received EUA in December 2020 in the United States [8, 9]. Billions of vaccine doses have been administered worldwide [10]. However, recognizing and tracking the side effects of COVID-19 vaccines is challenging, as some individuals have concerns about the vaccine’s safety [11]. Although clinical trials have ensured vaccine safety and efficacy, vaccines developed using the new mRNA and recombinant vaccine technology in a short period of time has raised concerns about their safety among the public.

Different studies have found common vaccine side effects, such as pain at the injection site, swelling, redness, fever, itching, headache, fatigue, joint and muscle pain, and night sweats [12–14]. However, it has been reported that side effects decreased after multiple-dose administration. Most importantly, there was a need to rapidly develop a vaccine to prevent the fast spread of the virus. Communities worldwide are protected from infectious diseases each year due to vaccination [15]. Therefore, the benefits of immunization outweigh the risks. Several pharmaceutical and biotechnology companies competed to be the first to develop and commercialize COVID-19 vaccines [16]. Pfizer-BioNTech, and Moderna quickly pioneered the development of vaccines using previously unapproved mRNAs. Pfizer-BioNTech was the first to cross the border in the US on December 11^th^, 2020, to obtain an EUA, and Moderna® announced the EUA a week later [17, 18]. The development process to commercialization, which normally takes 7–10 years, was completed in 10 months. A viral vector vaccine was also under development. Johnson & Johnson received the EUA of its vaccine in the USA in March 2021. AstraZeneca’s vaccine is also a viral vector and is in use in several countries, although some have decided to stop deploying it after reports of blood clots in vaccinated individuals [19]. Although most of the reported side effects are minor, people still believe in conspiracy theories and misinformation about the COVID-19 vaccines [10, 20]. This requires extensive research to explore the factors associated with higher risk of vaccines-related side effects. Potential predictors of vaccination side effects are age, gender, and ABO blood groups, among others [7, 21–24]. However, existing studies show variability in identifying predictors of the severity of COVID-19 vaccine-related side effects. In Saudi Arabia, Almalki et al conducted a nationwide survey to assess the relationships between such variables and the severity of adverse reactions. The researchers identified the education level and nationality of the participants following the first dose, gender following the second dose, and general health status after all of the doses as significant predictors of severe adverse reactions [20]. The study found no significant correlation between the severity of adverse vaccine-related reactions and ABO blood-type groups. In our study, we wanted to replicate the methodological approach used in the previous study of the general population by focusing on a special population consisting of university students to determine whether the previous findings would be supported.

The objectives of our present study were to identify the side effects that Taif University (TU) students reported after receiving COVID-19 vaccines; compare the side effects’ severity, onset, and duration by vaccine manufacturer; and identify the associated predictors.

## Materials and methods

### Settings and participants

This was a cross-sectional study conducted using a self-reported online questionnaire to investigate the predictors of severity of COVID-19 vaccine-related side effects in young population in the Kingdom of Saudi Arabia (KSA). A convenient sampling strategy was used to select students from TU living in the region from January 2022 to July 2022. The questionnaire was distributed to the students and their groups through social media using a link to the Google form. Also, the deanship of scientific research at TU emailed the questionnaire link to the students using official emails. The inclusion criteria included participants who had received at least one dose of COVID-19 vaccine of any type (*n* = 760). The study protocol was approved by the Research Ethics Committee of TU (Approval No. 43-172). All participants provided online consent for participation in the study.

### Study tool

A previously published validated questionnaire was used, which was administered in Arabic for optimal comprehension by the participants [25]. The questionnaire consisted of three sections. Section one included the demographic characteristics e.g., gender, age, nationality, social status, college, academic level, whether the participant has a family member who is working in the healthcare sector, and city of residence. Section two (seven questions) included questions about the clinical characteristics of the participants, such as student’s blood group, Rhesus (Rh) factor, self-reported general health status, COVID-19 vaccination status, history of COVID-19 infection, and the presence of comorbidities that mandate continuous administration of medication (e.g., asthma, diabetes, and hypertension). Section three was about self-reported COVID-19 vaccine-related side effects, including type, onset, duration, and severity after each of the three doses of the COVID-19 vaccine. Side effects’ severities were self-reported on a Likert scale from 1 to 10 and were classified as mild (1-3), moderate (4-7), and severe (8-10).

### Statistical analyses

Descriptive statistics were performed for the numerical and categorical variables to establish the frequency and percentage distribution of the demographic data and clinical characteristics. A chi-square test was performed for the cross-tabulation to the vaccine type and the severity of side effect-related variables and for investigating the correlation with other sociodemographic and clinical characteristics. A *p*-value of less than 0.05 was considered significant. All analyses were performed using the Statistical Package for Social Sciences (SPSS) software for Windows (Version 25.0, IBM Corp., Armonk, NY, USA).

## Results

A total of 760 adult participants from TU were included in the study. Most of them were 20–25 years old (79%, *n* = 600), 50.9% of them were females (*n* = 387), 97% (*n* = 737) were Saudi citizens, 42.8% (*n* = 325) were students in the medical colleges (Medicine, Dentistry, Pharmacy, and Applied Medical Sciences), 57.8% (*n* = 439) were between the 4^th^ to 6^th^ academic year, and 92.5% (*n* = 703) were from Taif. These data and other sociodemographic data are shown in Table 1.

**Table 1:** Demographic characteristics of 760 students who participated in the study

Characteristics	n (%)
**Age (years)**	
< 20	132 (17.4)
20–25	600 (78.9)
> 25	28 (3.7)
**Gender**	
Male	373 (49.1)
Female	387 (50.9)
**Nationality**	
Saudi	737 (97.0)
Non-Saudi	23 (3.0)
**Marital status**	
Single	719 (94.6)
Married	35 (4.6)
Divorced/Widow	6 (0.8)
**Current academic year**	
1–3	321 (42.2)
4 and above (including the internship year)	439 (57.8)
**College**	
-Medical colleges (medicine, pharmacy, applied medical science, dentistry)	325 (42.8)
-Scientific or engineering colleges (science, computers, engineering, design)	254 (33.4)
-College of humanities or educational sciences (arts, education, applied college, sharia-administrative sciences)	181 (23.8)
**Having a healthcare professional in the family**	
Yes	325 (42.8)
No	435 (57.2)
**City of residence**	
Taif	703 (92.5)
Makkah	32 (4.2)
Others	25 (3.3)

Regarding clinical characteristics, 40% (*n* = 304) had type O blood group. Rh factor was positive in 44.3% of the participants (*n* = 337), while it was unknown in 366 subjects (48.2%). Most of the participants (*n* = 476, 62.6%) received three doses of COVID-19 vaccine, while one-third were infected with COVID-19 since the start of the pandemic, of whom approximately 21% were infected after vaccination. The Pfizer-BioNTech vaccine was the first dose for 76.3% (*n* = 580) of them. Most of the participants (91.1%, *n* = 692) had no history of any chronic disease, and 706 of them (92.9%) reported having good health status (Table 2).

**Table 2:** Clinical characteristics of 760 students who participated in the study

Characteristics	n (%)
**Blood groups**	
A	180 (23.7)
B	100 (13.2)
AB	18 (2.4)
O	304 (40.0)
Don’t know	158 (20.8)
**Rh Factor**	
+ ve	337 (44.3)
-ve	57 (7.5)
Unknown	366 (48.2)
**History of COVID-19 infection since the pandemic**	
Yes	256 (33.7)
No	504 (66.3)
**History of COVID-19 Vaccination**	
None	0 (0.0)
First	760 (100)
Second	757 (99.6)
Third	476 (62.6)
**Self-reported general health status**	
Good	706 (92.9)
Fair	48 (6.3)
Poor	6 (0.8)
**Presence of chronic diseases**	
Yes	68 (8.9)
No	692 (91.1)
**Post- vaccine COVID-19 infection**	
Yes, but currently healthy	153 (20.1)
Yes, and currently infected	6 (0.8)
No	601 (79.1)
**COVID-19 vaccine type for first dose**	
Pfizer-BioNTech	580 (76.3)
AstraZeneca	160 (21.1)
Moderna	9 (1.2)
Unknown	11 (1.4)

The most frequent COVID-19 vaccine-related side effects after the first dose were pain at the injection site (54.7%), headache (45.0%), lethargy and fatigue (43.3%), and fever (37.5%). However, severe side effects rarely occurred after the first dose, such as low blood pressure (1.1%), heartbeat disturbance (2.6%), thrombosis (0.9%), and seizures (0.3%). The most frequent side effects after the second dose were pain at the injection site (49.7%), fatigue (48.5%), and fever (48.5%). Furthermore, the most frequent side effects after the third dose were pain at the injection site (49.6%), fatigue (47.7%), and fever (42.2%). Severe side effects were also rare after the second and third doses. Table 3 shows the frequency of each side effect for the three doses.

**Table 3:** Frequency of side effects post vaccination

Side effect	Post- 1^st^ dose	Post- 2^nd^ dose	Post- 3^rd^ dose
	**Frequency (%)**	**Frequency (%)**	**Frequency (%)**
	***n* = 760**	***n* = 757**	***n* = 476**
Abdominal discomfort	25 **(**3.3)	22 (2.9)	13 (2.7)
Palpitation	20 (2.6)	23 (3.0)	11 (2.3)
Chest pain	26 (3.4)	16 (2.1)	17 (3.6)
Injection site pain	416 (54.7)	376 (49.7)	236 (49.6)
Fever	285 (37.5)	367 (48.5)	201 (42.2)
Headache	342 (45.0)	329 (43.5)	183 (38.4)
Diarrhea	21 (2.8)	20 (2.6)	9 (1.9)
Sweating	40 (5.3)	37 (4.9)	17 (3.6)
Shivering	47 (6.2)	44 (5.8)	28 (5.9)
Joint pain	148 (19.5)	97 (12.8)	65 (13.7)
Dysmenorrhea or missed period	63 (8.3)	46 (6.1)	23 (4.8)
Hypotension	8 (1.1)	5 (0.7)	11 (2.3)
Thrombosis	7 (0.9)	6 (0.8)	4 (0.8)
Peripheral edema	20 (2.6)	17 (2.3)	9 (1.9)
Lethargy/ fatigue	329 (43.3)	367 (48.5)	227 (47.7)
Cold extremities	39 (5.1)	36 (4.8)	26 (5.5)
Numbness	39 (5.1)	36 (4.8)	26 (5.5)
Skin rash/itchiness	22 (2.9)	21 (2.8)	10 (2.1)
Rhinorrhea/nasal congestion	36 (4.7)	30 (4.0)	23 (4.8)
Shortness of breath	53 (7.0)	46 (6.1)	29 (6.1)
Visual disturbance	20 (2.6)	19 (2.5)	9 (1.9)
Nausea/vomiting	41 (5.4)	39 (5.2)	20 (4.2)
Dizziness	34 (4.5)	29 (3.8)	17 (3.6)
Seizures	2 (0.3)	4 (0.5)	1 (0.2)
Nasal/gingival bleed	4 (0.5)	5 (0.7)	1 (0.2)

The reported percentage is column percentage

Receiving the Pfizer-BioNTech vaccine resulted in more mild-to-moderate side effects compared to those of the other manufacturers (Table 4). This was found separately after the first, second, and third doses given that a respondent may have received doses from various manufacturers. However, slightly less than half of all respondents who reported the types of vaccine (48.84%) received all three doses from Pfizer-BioNTech, and the majority (81.2%) received at least two doses from Pfizer-BioNTech. This finding remained the same when analyses included those who received vaccines in the second and third doses from the same manufacturers compared to those who received mixed types (Supplemental Table 1). Moreover, side effects usually started within 8 hours of receiving each dose of the three types of vaccines used in KSA and lasted for 1–3 days, with significant differences between the three vaccine types.

**Table 4:** Characteristics of COVID 19 vaccine related side effects by manufacturer

Item/ Vaccine			AstraZeneca n (%)	Pfizer-BioNTech n (%)	Moderna n (%)	Total n (%)	*P*-value^#^
Side effects after 1^st^ Dose^a^	Severity	Mild	30 (18.8)	248 (42.8)	2 (22.2)	280 (37.4)	0.001
		Moderate	64 (40.0)	223 (38.5)	5 (55.6)	292 (39.0)	
		Severe	60 (37.5)	72 (12.4)	2 (22.2)	134 (17.9)	
		Not reported	6 (3.75)	37 (6.4)	0 (0)	43 (5.7)	
	Onset	0–8 h	91 (56.9)	324 (55.9)	2 (22.2)	417 (55.7)	0.001
		9–24 h	53 (33.1)	148 (25.5)	7 (77.8)	208 (27.8)	
		Not reported	16 (10.0)	108 (18.6)	0 (0)	124 (16.6)	
	Duration	< 1 day	45 (28.1)	120 (20.7)	4 (44.4)	169 (22.6)	0.018
		1 – 3 days	83 (51.9)	306 (52.8)	5 (55.6)	394 (52.6)	
		> 3 days	17 (10.6)	45 (7.8)	0 (0)	62 (8.3)	
		Not reported	15 (9.4)	109 (18.8)	0 (0)	124 (16.6)	
	Total		160 (21.4)	580 (77.4)	9 (1.2)	749 (100)	
Side effects after 2^nd^ Dose^a^	Severity	Mild	51 (35.4)	230 (42.3)	6 (15.4)	287 (39.5)	0.001
		Moderate	43 (29.9)	182 (33.5)	17 (43.6)	242 (33.3)	
		Severe	44 (30.6)	97 (17.8)	16 (41.0)	157 (21.6)	
		Not reported	6 (4.2)	35 (6.4)	0 (0)	41 (5.6)	
	Onset	0–8 h	83 (57.6)	286 (52.6)	27 (69.2)	396 (54.5)	0.091
		9–24 h	33 (22.9)	143 (26.3)	9 (23.1)	185 (25.5)	
		Not reported	28 (19.5)	115 (21.1	3 (7.7)	146 (20.0)	
	Duration	< 1 day	37 (25.7)	135 (24.8)	9 (23.1)	181 (24.9)	0.212
		1 – 3 days	63 (43.8)	249 (45.8)	25 (64.1)	337 (46.4)	
		> 3 days	16 (11.1)	47 (8.6)	3 (7.7)	66 (9.1)	
		Not reported	28 (19.4)	113 (20.8)	2 (5.1)	143 (19.7)	
	Total		144 (19.8)	544 (74.8)	39 (5.4)	727 (100)	
Side effects after 3^rd^ Dose^a^	Severity	Mild	5 (45.5)	140 (39.0)	31 (29.2)	176 (37.0)	0.001
		Moderate	2 (18.2)	122 (34.0)	29 (27.4)	153 (32.1)	
		Severe	3 (27.3)	67 (18.7)	41 (38.7)	111 (23.3)	
		Not reported	1 (9.1)	30 (8.4)	5 (4.7)	36 (7.6)	
	Onset	0–8 h	6 (54.5)	172 (47.9)	57 (53.7)	235 (49.4)	0.546
		9–24 h	1 (9.1)	97 (27.0)	31 (29.3)	129 (27.1)	
		Not reported	4 (36.4)	90 (25.1)	18 (17.0)	112 (23.6)	
	Duration	< 1 day	3 (27.3)	78 (21.7)	23 (21.7)	104 (21.9)	0.725
		1 – 3 days	4 (36.4)	158 (44.0)	50 (47.2)	212 (44.5)	
		> 3 days	0 (0.0)	32 (8.9)	14 (13.2)	46 (9.7)	
		Not reported	4 (36.4)	91 (25.3)	19 (17.9)	114 (23.9)	
	Total		11 (2.3)	359 (75.4)	106 (22.3)	476 (100)	

^a^Percentage calculated for those who reported vaccine manufacturer

^#^Chi-square test

Table 5 shows the correlation between the severity of the vaccine-related side effects and the demographic characteristics of the study participants. The finding showed that the most frequent side effects were reported among the 20–25-year age group for all doses of all types of vaccines. Side effects were significantly more among females after the second (*p* < 0.001) and third doses (*p* = 0.005). Also, nationality showed a significant correlation with COVID-19 vaccine-related side effects after the first dose of the vaccine (*p* = 0.043). Additionally, the educational level showed a significant correlation with COVID-19 vaccine-related side effects, with a higher frequency in those in the 4^th^ to 6^th^ academic year, especially after the first dose (*p* = 0.008).

**Table 5:** Correlation between severity of vaccine-related side effects and participants’ demographic characteristics

Demographic characteristics		First dose (*n* = 717)^a^			p^#^	Second dose (*n* = 718)^a^			p^#^	Third dose (*n* = 440)^a^			P^#^
		Mild	Moderate	Severe		Mild	Moderate	Severe		Mild	Moderate	Severe	
Age^a^ (years)	< 20	58 (20.3)	55 (18.8)	15 (10.9)	0.069	43 (14.4)	58 (22.6)	26 (16.0)	0.070	19 (10.8)	34 (22.2)	20 (18.0)	0.054
	20–25	216 (75.5)	231 (78.8)	115 (83.3)		241 (80.9)	193 (75.1)	129 (79.1)		149 (84.7)	113 (73.9)	89 (80.2)	
	> 25	12 (4.2)	7 (2.4)	8 (5.8)		14 (4.7)	6 (2.3)	8 (4.9)		8 (4.6)	6 (3.9)	2 (1.8)	
	Total	286 (39.9)	293 (40.9)	138 (19.2)		298 (41.5)	257 (35.8)	163 (22.7)		176 (40.0)	153 (34.8)	111 (25.2)	
Gender^a^	Male	146 (51.0)	131 (44.7)	68 (49.3)	0.298	170 (57.0)	109 (42.4)	67 (41.1)	0.001	96 (54.6)	75 (49.0)	39 (35.1)	0.005
	Female	140 (49.0)	162 (55.3)	70 (50.7)		128 (43.0)	148 (57.6)	96 (58.9)		80 (45.6)	78 (51.0)	72 (64.9)	
	Total	286 (39.9)	293 (40.9)	138 (19.2)		298 (41.5)	257 (35.8)	163 (22.7)		176 (40.0)	153 (34.8)	111 (25.2)	
Nationality^a^	Saudi	282 (98.6)	282 (96.2)	130 (94.2)	0.043	290 (97.3)	247 (96.1)	158 (96.9)	0.719	170 (96.6)	146 (95.4)	109 (98.2)	0.472
	Non-Saudi	4 (1.4)	11 (3.8)	8 (5.8)		8 (2.7)	10 (3.9)	5 (3.1)		6 (3.4)	7 (4.6)	2 (1.8)	
	Total	286 (39.9)	293 (40.9)	138 (19.2)		298 (41.5)	257 (35.8)	163 (22.7)		176 (40.0)	153 (34.8)	111 (25.2)	
Marital status^a^	Single	270 (94.4)	277 (94.5)	130 (94.2)	0.950	283 (95.0)	241 (93.8)	154 (94.5)	0.800	166 (94.3)	144 (94.1)	107 (96.4)	0.599
	Married	14 (4.9)	13 (4.4)	7 (5.1)		13 (4.3)	14 (5.4)	7 (4.3)		8 (4.6)	8 (5.2)	3 (2.7)	
	Divorced/Widow	2 (0.7)	3 (1.1)	1 (0.7)		2 (0.7)	2 (0.8)	2 (1.2)		2 (1.1)	1 (0.7)	1 (0.9)	
	Total	286 (39.9)	293 (40.9)	138 (19.2)		298 (41.5)	257(35.8)	163 (22.7)		176 (40.0)	153 (34.8)	111 (25.2)	
Current academic year^a^	1^st^ -3^rd^	137 (47.9)	125 (42.7)	44 (31.9)	0.008	126 (42.3)	121 (47.1)	62 (38.0)	0.178	76 (43.2)	67 (43.8)	52 (46.8)	0.820
	4^th^ -6^th^	149 (52.1)	168 (57.3)	94 (68.1)		172 (57.7)	136 (52.9)	101 (62.0)		100 (56.8)	86 (56.2)	59 (53.2)	
	Total	286 (39.9)	293 (40.9)	138 (19.2)		298 (41.5)	257 (35.8)	163 (22.7)		176 (40.0)	153 (34.8)	111 (25.2)	
College^a^	Medical colleges	127 (44.4)	130 (44.4)	52 (37.7)	0.634	133 (44.6)	106 (41.2)	66 (40.5)	0.550	81 (46.0)	66 (43.1)	46 (41.4)	0.461
	Scientific or engineering colleges	95 (33.2)	91 (31.1)	49 (35.5)		102 (34.2)	84 (32.7)	52 (31.9)		62 (35.2)	46 (30.1)	40 (36.0)	
	Humanities or educational sciences	64 (22.4)	72 (24.6)	37 (26.8)		63 (21.1)	67 (26.1)	45 (27.6)		33 (18.8)	41 (26.8)	25 (22.5)	
	Total	286 (39.9)	293 (40.9)	138 (19.2)		298 (41.5)	257 (35.8)	163 (22.7)		176 (40.0)	153 (34.8)	111 (25.2)	
HCW^b,a^	Yes	116(40.6)	132 (45.1)	58 (42.0)	0.543	121 (40.6)	115 (44.7)	67 (41.1)	0.584	73 (41.5)	73 (47.7)	58 (52.3)	0.187
	No	170 (59.4)	161 (54.4)	80 (58.0)		177 (59.4)	142 (55.3)	96 (58.9)		103 (58.5)	80 (52.3)	53 (47.7)	
	Total	286 (39.9)	293 (40.9)	138 (19.2)		298 (41.5)	257 (35.8)	163 (22.7)		176 (40.0)	153 (34.8)	111 (25.2)	
City of residence^a^	Taif	268 (93.7)	273 (93.2)	123 (89.1)	0.338	273 (91.6)	237 (92.2)	153 (92.3)	0.925	164 (93.2)	135 (88.2)	105 (94.6)	0.315
	Makkah	8 (2.8)	13 (4.4)	8 (5.8)		15 (5.0)	11 (4.3)	6 (3.7)		7 (4.0)	9 (5.9)	4 (3.6)	
	Others	10 (3.5)	7 (2.4)	7 (5.1)		10 (3.4)	9 (3.5)	4 (2.5)		5 (2.8)	9 (5.9)	2 (1.8)	
	Total	286 (39.9)	293 (40.9)	138 (19.2)		298 (41.5)	257 (35.8)	163 (22.7)		176 (40.0)	153 (34.8)	111 (25.2)	

^a^Percentage calculated based on those who reported any side effect(s)

^b^Healthcare workers in the family

^#^Chi-square test

ABO blood groups showed a significant correlation with vaccine-related side effects after the second dose (*p* = 0.020). Also, the general health status of the participants showed a significant correlation with vaccine-related side effects after the first (*p* < 0.001) and second doses of the vaccines (*p* = 0.022). However, the Rh factor or the presence of chronic diseases showed no correlation with vaccine-related side effects after the three doses of the vaccine (Table 6).

**Table 6:** Correlation between severity of vaccine-related side effects and participants’ clinical characteristics

Clinical character		First dose			p^#^	Second dose			p^#^	Third dose			p^#^
		Mild	Moderate	Severe		Mild	Moderate	Severe		Mild	Moderate	Severe	
Blood group^a^	A	73 (30.7)	67 (31.5)	29 (25.2)	0.245	82 (33.3)	53 (26.1)	34 (28.8)	0.020	44 (29.5)	34 (27.6)	32 (37.2)	0.808
	B	34 (14.3)	33 (15.5)	28 (24.3)		25 (10.2)	44 (21.7)	26 (22.0)		25 (16.8)	23 (18.7)	13 (15.1)	
	AB	6 (2.5)	5 (2.3)	5 (4.3)		9 (3.7)	5 (2.5)	2 (1.7)		4 (2.7)	5 (4.1)	2 (2.3)	
	O	125 (52.5)	108 (50.7)	53 (46.1)		130 (52.8)	101 (49.8)	56 (47.5)		76 (51.0)	61 (49.6)	39 (45.3)	
	Total	238 (40.1)	213 (37.6)	115 (20.3)		246 (43.4)	203 (35.8)	118 (20.8)		149 (41.6)	123 (34.4)	86 (24.0)	
Rh Factor^a^	+ ve	127 (84.7)	117 (87.3)	66 (82.5)	0.616	132 (84.6)	115 (86.5)	63 (84.0)	0.862	74 (82.2)	75 (84.3)	46 (85.2)	0.881
	- ve	23 (15.3)	17 (12.7)	14 (17.5)		24 (15.4)	18 (13.5)	12 (16.0)		16 (17.8)	14 (15.7)	8 (14.8)	
	Total	150 (41.2)	134 (36.8)	80 (22.0)		156 (42.9)	133 (36.5)	75 (20.6)		90 (38.6)	89 (38.2)	54 (23.2)	
Infection status since pandemic started^a^	Yes	93 (32.5)	94 (32.1)	53 (38.4)	0.391	104 (34.9)	77 (30.0)	58 (35.6)	0.365	56 (31.8)	40 (26.1)	37 (33.3)	0.381
	No	193 (67.5)	199 (67.9)	85 (61.6)		194 (65.1)	180 (70.0)	105 (64.4)		120 (68.2)	113 (73.9)	74 (66.7)	
	Total	286 (39.9)	293 (40.9)	138 (19.2)		298 (41.5)	257 (35.8)	163 (22.7)		176 (40.0)	153 (34.8)	111 (25.2)	
General health status^**a**^	Good	276 (96.5)	271 (92.5)	117 (84.8)	0.001	283 (95.0)	236 (91.8)	146 (89.6)	0.022	169 (96.0)	141 (92.2)	104 (93.7)	0.390
	Fair	10 (3.5)	21 (7.2)	16 (11.6)		13 (4.3)	21 (8.2)	13 (8.0)		7 (4.0)	10 (6.5)	5 (4.5)	
	Poor	0 (0.0)	1 (0.3)	5 (3.6)		2 (0.7)	0 (0.0)	4 (2.4)		0 (0.0)	2 (1.3)	2 (1.8)	
	Total	286 (39.9)	293 (40.9)	138 (19.2)		298 (41.5)	257 (35.8)	163 (22.7)		176 (40.0)	153 (34.8)	111 (25.2)	
Chronic diseases^a^	Yes	20 (7.0)	33 (11.3)	13 (9.4)	0.205	30 (10.1)	28 (10.9)	9 (5.5)	0.155	15 (8.5)	13 (8.5)	9 (8.1)	0.991
	No	266 (93.0)	260 (88.7)	125 (90.6)		268 (89.9)	229 (89.1)	154(94.5)		161 (91.5)	140 (91.5)	102 (91.9)	
	Total	286 (39.9)	293 (40.9)	138 (19.2)		298 (41.5)	257 (35.8)	163 (22.7)		176 (40.0)	153 (34.8)	111 (25.2)	
Infection status after vaccination^a^	Yes	57 (20.4)	62 (21.2)	26 (19.4)	0.899	66 (23.0)	46 (19.0)	29 (18.5)	0.273	31 (17.6)	26 (17.0)	21 (18.9)	0.179
	No	223 (79.6)	230 (78.8)	108 (80.6)		221 (77.0)	196 (81.0)	128 (81.5)		145 (82.4)	127 (83.0)	90 (81.1)	
	Total	280 (39.7)	292 (41.4)	134 (19.0)		287 (41.8)	242 (35.3)	157 (22.9)		176 (40.0)	153 (34.8)	111 (25.2)	

^a^Percentage calculated based on having answers for both tested variables

^#^Chi-square test

Multivariate regression analysis shows a significant correlation between the severity of the vaccine-related side effects and female gender (*p* = 0.025), vaccine type (*p* = 0.014 for AstraZeneca), poor general health status (*p* = 0.012) and Blood group B (*p* = 0.017 for severe side effects, Table 7).

**Table 7:** Categories having higher risk for developing severe side effects after receiving COVID-19 vaccine

	**Severe**	
	**aOR (95% CI)**	***p* value**^#^
**Age group**		
> 25 y	1	
< 25 y	2.16 (0.67–6.96)	0.198
**Gender**		
Male	1	
Female	2.14 (1.04–6.16)	0.025
**Nationality**		
Saudi	1	
Non-Saudi	2.35 (0.69–10.71)	0.136
**Marital status**		
Married	1	
Single	1.77 (0.64–4.86)	0.721
**Current academic year**		
1^st^ – 3^rd^ year	1	
4^th^ – and above	0.88 (0.41–1.79)	0.529
**College**		
Medical	1	
Engineering	0.69 (0.19–5.88)	0.254
Arts	0.48 (0.07–4.39)	0.317
**Type of vaccine**		
Moderna	1	
AstraZeneca	1.86 (0.41–2.88)	0.014
Pfizer-BioNTech	1.13 (0.57–9.51)	0.305
**City of residence**		
Makkah	1	
Taif	0.28 (0.11–3.88)	0.253
Other	0.39 (0.23–3.78)	0.641
**Blood type**		
A	1	
B	1.91 (0.81–3.21)	0.017
AB	1.17 (0.89–5.71)	0.356
O	1.41 (0.88–3.18)	0.462
Unknown	0.63 (0.29–4.67)	0.783
**Rh Factor**		
Positive	1	
Negative	0.41 (0.29–5.31)	0.358
Unknown	0.87 (0.37–7.36)	0.417
**Self-reported general health status**		
Good	1	
Fair	1.81 (0.73–3.39)	0.045
Poor	1.97 (1.06–4.68)	0.012
**Presence of chronic diseases**		
No	1	
Yes	1.62 (0.96–5.87)	0.064
**Post-vaccination COVID-19 infection**		
No	1	0.169
Yes	0.73 (0.46–5.14)	

*aOR* Adjusted odds ratio

^#^Chi-square test

## Discussion

This study sought to identify predictors of the severity of side effects after COVID-19 vaccines among young adult students at Taif University (TU). Since the pandemic, several COVID-19 vaccines have been licensed [17], and the success of a launch often depends on people’s willingness to accept it considering the highlighted safety profile, taking into account the fact that the development of vaccines and the technology used utilized either mRNA technology or a virus belonging to the adenovirus family, genetically modified with a gene encoding a specific SARS-CoV-2 protein [26]. This study indicated that the severity of COVID-19 vaccine-related side effects depend on several factors, such as the COVID-19 vaccine type, gender, ABO blood groups, age, and general health status.

The study results suggested that both females and males experienced side effects that mostly started within 8 hours after vaccination, lasted between one and three days, and were mainly limited to pain at the injection site, headache, fatigue, and fever, while serious side effects were rare. These results are consistent with what has been reported in the literature [27, 28]. In this study, we also found that both females and males reported side effects to the vaccine over the three doses, but females showed more side effects that were between moderate and severe than their male counterparts. These findings are in agreement with previous international studies reporting fewer side effects in males, which could be attributed to psychological variation between genders [29, 30].

Our study did not find any correlation between the severity of side effects and chronic illnesses. This observation could be attributed to the fact that the study participants were young and that most of them were free from chronic illnesses. Interestingly, we found that being of blood group B, receiving AstraZeneca vaccine, and being in poor general health were significant predictors of the occurrence and severity of vaccine-related side effects. The results were in partial disagreement with two local studies, one by Alessa et al., which included 612 surgeons, and one by Almalki et al., which included 1180 adult participants in the community across KSA, and neither study reported an association between the appearance or severity of COVID-19 vaccine-related side effects and blood type [7, 20]. However, both studies supported our finding that the severity of side effects was associated with gender and vaccine type. It is worth noting that Alessa et al. and Almalki et al. included older and broader age groups than those in our study whereas we recruited students from Taif University who resided in the cities of Taif and Makkah, with about 95% of the participants in our study being younger than 25 years old. Also, most of the students enrolled herein are from Taif and Makkah (~97%), and they differ in marital status, education level, and general health status whereas more than 40% of the participants in Almalki et al. study [20], were from other cities of Saudi Arabia and differ in demographic characteristics, which could lead to such conflicting findings in the two studies. On the other hand, a history of COVID-19 infection was not a predictor of the occurrence or severity of COVID-19 vaccine-related side effects compared to non-infected participants. These results are consistent with those Alessa et al. [7] and Almalki et al. [20] reported.

On the other hand, our results contradict what was reported by Beatty et al. [30], who found that people with past COVID-19 history before vaccination had greater odds of developing adverse effects or more severe forms of adverse effects after COVID-19 vaccination.

Our study found that being of blood group B was a predictor of developing more severe forms of side effects after vaccination. It is also possible to read such findings from the opposite perspective, saying: group A individuals were less susceptible to severe side effects of the vaccine. To our knowledge, this was the first study to discern this association, compared to other local studies that included broader and older age groups [7, 25], and to those conducted internationally. In this regard, according to growing data, the ABO blood group system could be involved in the immuno-pathogenesis of SARS-CoV-2 infection, with blood group O people being less likely to test positive and group A and B individuals having both a higher sensitivity to infection and a tendency toward severe disease. Also, subjects of blood group A may have unfavorable prognoses. In addition, several observational studies, genome-wide association reports, and country-level meta-regression analyses all provide evidence of a link between ABO group and susceptibility to SARS-CoV-2/COVID-19 infection, as reviewed elsewhere [31–34]. Though it was not evident in Almalki et al.’s study, the relationship our study found between blood group and severity of vaccine side effects might be linked to certain age groups (i.e., revealing a possible interaction between age and blood groups). The role of age as a moderator for other variables effects is well known and widely hypothesized, tested, or proven in medical literature [28, 29]. However, we could not determine whether Almalki et al’s study did not find such a relationship because the relationship was not present at all or was present but could not be detected due to the small sample size. The latter option is particularly possible because the former study included a wider range of variable ages, and the significance of the relationship is only detectable when the pattern differs more between different groups than within groups. In our current study, the age variations are much smaller, with all participants being aged between 18 to 28.

This study has some limitations due to the nature of self-reporting and its cross-sectional design. Participants may over- or underestimate their self-reported side effects. There is evidence from literature supports an existence of a nocebo response, which can be defined as a negative reaction or symptom that is believed to be reported because of an individual’s expectation of developing negative events after receiving a medical intervention such as a drug or vaccine [35, 36]. However, it is to be noted that a large percentage of our participants did not report any side effects and the reported side effects were low in frequency in most cases, nullifying the presence of nocebo effect. The cross-sectional design cannot assess the temporal relationships and prospective follow-up studies will be needed to confirm findings. Also, we included only TU students, which may limit the generalizability of the study to others. In addition, other factors that might affect the development and severity of the vaccine-related side effects, such as ethnicity, pregnancy, history of asthma at baseline, receiving an influenza shot in the previous year, and social status, were not addressed in the study.

This study provided real-world data about predictors of COVID-19 vaccine-related side effects across three brands of COVID-19 vaccines (AstraZeneca, Moderna, and Pfizer-BioNTech) in young, vaccinated people in KSA for the three doses. Also, we were able to investigate predictors of COVID-19 vaccine-related side effects in a more focused special population as we included young participants who were between 18 and 26 years old to explore the age’s effect on the appearance and severity of COVID-19 vaccine-related side effects. Future studies with larger sizes and more diverse population characteristics are needed to confirm the relationship between blood group and side effect severity, explore the mechanism therein, and test the age–blood group interaction we hypothesize.

## Conclusions

Blood group B, female gender, vaccine type, and poor health status were among the predictors of COVID-19 vaccine-related side effects in young COVID-19 vaccinated people in KSA. Future national digital post-marketing surveillance for COVID-19 vaccine-related side effects should be conducted to better understand the role of ABO blood groups and other predictors in the safety of COVID-19 vaccines in young vaccinated individuals due to the inconsistency in the literature in reporting predictors of COVID-19 vaccine-related side effects.

## Supplementary Information

**Additional file 1:** **Supplemental Table 1. **Characteristics of COVID 19 vaccine related side effects by manufacturer.

## Data Availability

The raw data supporting the conclusions of this article will be made available by the authors, without undue reservation at osmalki@tu.edu.sa. Competing interests: The authors declare no competing interests.
