# Supplementary material for: The role of blood groups, vaccine type and gender in predicting the severity of side effects among university students receiving COVID-19 vaccines
Source: BMC Infect Dis. 2023 Jun 6;23:378. doi: 10.1186/s12879-023-08363-0 (PMC10242595; doi:10.1186/s12879-023-08363-0)
Supplement: Supplementary file 1 — Additional file 1: Supplemental Table 1. Characteristics of COVID 19 vaccine related side effects by manufacturer. [file 12879_2023_8363_MOESM1_ESM.docx]

**Supplemental Table 1:** Characteristics of COVID 19 vaccine related side effects by manufacturer

| **Item/ Vaccine** | | | **AstraZeneca**  **n (%)** | **Pfizer-BioNTech**  **n (%)** | **Moderna**  **n (%)** | **Mixed vaccine types**  **n (%)** | **Total**  **n (%)** | **P-value** |
| --- | --- | --- | --- | --- | --- | --- | --- | --- |
| **Side effects after 1^st^ Dose**^a^ | **Severity** | Mild  Moderate  Severe  Not reported | 30 (18.8)  64 (40.0)  60 (37.5)  6 (3.75) | 248 (42.8)  223 (38.5)  72 (12.4)  37 (6.4) | 2 (22.2)  5 (55.6)  2 (22.2)  0 (0) | - | 280 (37.4)  292 (39.0)  134 (17.9)  43 (5.7) | 0.001 |
|  | **Onset** | 0-8 hours  9-24 hours  Not reported | 91 (56.9)  53 (33.1)  16 (10.0) | 324 (55.9)  148 (25.5)  108 (18.6) | 2 (22.2)  7 (77.8)  0 (0) | - | 417 (55.7)  208 (27.8)  124 (16.6) | 0.001 |
|  | **Duration** | < 1 day  1 – 3 days  >3 days  Not reported | 45 (28.1)  83 (51.9)  17 (10.6)  15 (9.4) | 120 (20.7)  306 (52.8)  45 (7.8)  109 (18.8) | 4 (44.4)  5 (55.6)  0 (0)  0 (0) | - | 169 (22.6)  394 (52.6)  62 (8.3)  124 (16.6) | 0.018 |
|  | **Total** |  | **160 (21.4)** | **580 (77.4)** | **9 (1.2)** |  | **749 (100)** |  |
| **Side effects after 2^nd^ Dose**^a^ | **Severity** | Mild  Moderate  Severe | 14 (23)  20 (32.8)  27 (44.3) | 189 (45.1)  180 (43)  50 (11.9) | Not included  in analysis** | 77 (34.1)  92 (40.7)  57 (25.2) | 280 (39.7)  292 (41.4)  134 (19) | <0.001 |
|  | **Total** |  | 61 (100) | 419 (100) |  | 226 (100) | 706 (100) |  |
|  | **Onset** | 0-8 hours  9-24 hours  Not reported | 40 (63.5)  16 (25.4)  7 (11.1) | 245 (54.7)  122 (27.2)  81 (18.1) | Not included  in analysis** | 132 (55.5)  70 (29.4)  36 (15.1) | 417 (55.7)  208 (27.8)  124 (16.6) | 0.520 |
|  | **Total** |  | 63 (100) | 448 (100) |  | 238 (100) | 749 (100) |  |
|  | **Duration** | < 1 day  1 – 3 days  > 3 days  Not reported | 21 (33.3)  29 (46)  6 (9.5)  7 (11.1) | 94 (21)  240 (53.6)  32 (7.1)  82 (18.3) | Not included  in analysis** | 54 (22.7)  125 (52.5)  24 (10.1)  35 (14.7) | 169 (22.6)  394 (52.6)  62 (8.3)  124 (16.6) | 0.202 |
|  | **Total** |  | 63 (100) | 448 (100) |  | 238 (100) | 749 (100) |  |
| **Side effects after 3^rd^ Dose**^a^ | **Severity** | Mild  Moderate  Severe | Not included  in analysis* | 88 (41.7)  84 (39.8)  39 (18.5) | Not included  in analysis** | 87 (38.2)  69 (30.3)  72 (31.6) | 175 (39.9)  153 (34.9)  111 (25.3) | 0.005 |
|  | **Total** |  |  | 211 (100) |  | 228 (100) | 439 (100) |  |
|  | **Onset** | 0-8 hours  9-24 hours  None reported | Not included  in analysis* | 114 (51.8)  64 (29.1)  42 (19.1) | Not included  in analysis** | 120 (50.4)  65 (27.3)  53 (22.3) | 234 (51.1)  129 (28.2)  95 (20.7) | 0.695 |
|  | **Total** |  |  | 220 (100) |  | 238 (100) | 458 (100) |  |
|  | **Duration** | < 1 day  1 – 3 days  > 3 days  None reported | Not included  in analysis* | 54 (24.3)  106 (47.7)  19 (8.6)  43 (19.4) | Not included  in analysis** | 49 (20.5)  106 (44.4)  27 (11.3)  57 (23.8) | 103 (22.3)  212 (46)  46 (10)  100 (21.7) | 0.396 |
|  | **Total** |  |  | 222 (100) |  | 239 (100) | 461 (100) |  |
|  |  |  |  |  |  |  |  |  |

^a^ Percentage calculated for those who reported vaccine manufacturer

* Very few individuals, so they were excluded from the analysis

** Very few individuals, so they were excluded from the analysis
